# Regulation of 2,4-diacetylphloroglucinol biosynthesis and biocontrol capacity by the BolA family protein IbaG in *Pseudomonas fluorescens* 2P24

**DOI:** 10.1128/spectrum.00985-23

**Published:** 2023-09-19

**Authors:** Qiuling Dong, Qing Yan, Bo Zhang, Li-qun Zhang, Xiaogang Wu

**Affiliations:** 1 Guangxi Key Laboratory of Agro-Environment and Agro-Product Safety/College of Agriculture, Guangxi University, Nanning, China; 2 Department of Plant Sciences and Plant Pathology, Montana State University, Bozeman, Montana, USA; 3 College of Plant Protection, China Agricultural University, Beijing, China; Connecticut Agricultural Experiment Station, New Haven, Connecticut, USA

**Keywords:** *Pseudomonas fluorescens*, 2,4-DAPG, monothiol glutaredoxin GrxD, BolA, IbaG

## Abstract

The monothiol glutaredoxin GrxD plays an essential role in the biosynthesis of the antibiotic 2,4-diacetylphloroglucinol (2,4-DAPG) and the biocontrol capacity of the soil bacterium *Pseudomonas fluorescens* 2P24. However, the detailed mechanism underlying GrxD-mediated activation of the production of 2,4-DAPG remains unclear. Here, we found that GrxD directly interacted with IbaG, a BolA protein family member. The mutation of *ibaG* significantly decreased 2,4-DAPG production. Furthermore, expressing *ibaG* restored the production of 2,4-DAPG in the *grxD ibaG* double mutant to wild-type levels in the presence of dithiothreitol, suggesting that IbaG was required for GrxD-mediated regulation of 2,4-DAPG production. Transcriptome sequencing analyses revealed that IbaG plays a global role in gene regulation by affecting the expression of numerous genes throughout the genome. We also demonstrated that IbaG is an important regulator of several cellular processes, including swarming motility, biofilm formation, siderophore production, and acid resistance. Altogether, our data suggest that IbaG has an essential role in 2,4-DAPG production, motility, and biofilm formation. We also propose a regulatory mechanism linking GrxD to 2,4-DAPG production via IbaG.

**IMPORTANCE:**

The production of 2,4-diacetylphloroglucinol (2,4-DAPG) is positively influenced by the monothiol glutaredoxin GrxD in *Pseudomonas fluorescens* 2P24. However, the regulatory mechanism underlying GrxD-mediated regulation of 2,4-DAPG biosynthesis is mostly uncharacterized. Here, we show the function of the BolA-like protein IbaG in 2,4-DAPG biosynthesis. We also demonstrate that GrxD directly interacts with IbaG and influences the redox state of IbaG. Altogether, this work provides new insights into the role of the highly conserved IbaG protein in regulating 2,4-DAPG synthesis, biofilm formation, and other biocontrol traits of *P. fluorescens*.

## INTRODUCTION

*Pseudomonas fluorescens* belongs to a group of ubiquitous beneficial rhizobacteria that play important roles in inhibiting the growth of phytopathogens, promoting plant growth, and eliciting plant immune responses (1). The production of secondary metabolites with antibiotic activity is crucial for *P. fluorescens* to exert its biocontrol functions. Among these secondary metabolites, 2,4-diacetylphloroglucinol (2,4-DAPG) has received special attention because of its broad-spectrum antimicrobial activity (2). The gene cluster involved in 2,4-DAPG biosynthesis comprises the *phlACBD* operon. Specifically, the *phlD* gene encodes a type III polyketide synthase, which catalyzes the formation of phloroglucinol (PG) from malonyl-CoA. Monoacetylphloroglucinol (MAPG) acetyltransferase, which is encoded by *phlACBD*, acetylates PG to MAPG and 2,4-DAPG (3).

The biosynthesis of 2,4-DAPG is regulated by biotic and abiotic factors in response to various environmental cues. The pathway-specific regulators PhlF and PhlH are TetR-like repressors that inhibit the transcription of *phlACBD* and *phlG*, respectively. They bind directly to their target promoter region to regulate 2,4-DAPG metabolism (2, 4). Additionally, the Gac/Rsm signaling cascade plays a vital role in regulating 2,4-DAPG biosynthesis in *P. fluorescens* at the post-transcriptional level (5). The GacS/GacA two-component system induces the transcription of small regulatory RNAs, e.g., RsmX, RsmX1, RsmY, and RsmZ, which have high affinity for the CsrA protein family (6). In *Pseudomonas* spp., the members of the CsrA family, RsmA and RsmE, interact directly with their target mRNAs to affect mRNA stability or alter translational efficiency. It has been hypothesized that the distribution of a conserved 5′-CANGGAYG-3′ sequence motif, which overlaps with the ribosome binding site, is essential for RsmA/CsrA proteins to bind their target mRNA and regulate translation (7). For example, a previous study has shown that RsmA binds directly to *phlA* mRNA and influences its expression (6). Finally, various carbohydrate compounds, low temperatures, and metabolite exudates released by fungal hyphae and plant roots affect the production of 2,4-DAPG. In *P. protegens* Pf-5, the production of 2,4-DAPG is co-regulated with the biosynthesis of another antibiotic called pyoluteorin by PG (8).

*P. fluorescens* 2P24 was originally isolated from take-all decline soil. It suppresses a variety of soilborne diseases caused by plant pathogens, including *Rhizoctonia solani*, *Gaeumannomyces graminis* var. *tririci*, and *Ralstonia solanacearum* (9). Genetic analyses showed that 2,4-DAPG is the key biocontrol component of strain 2P24. Additionally, strain 2P24 is an efficient root colonizer that can durably colonize plant rhizosphere. Root colonization is a complicated process involving interactions between plant growth-promoting rhizobacteria, phytopathogens, and host plants (10). Reactive oxygen species produced by plant cells negatively affect rhizosphere colonization by *P. fluorescens*. Recently, we showed that the monothiol glutaredoxin GrxD is responsible for 2,4-DAPG production, oxidative stress tolerance, and other biocontrol traits of strain 2P24 (11). GrxD is involved in the biosynthesis of iron-sulfur (Fe-S) clusters and the reduction of thiol-disulfide exchange reaction in a glutathione-dependent manner. The conserved monothiol motif (CGFS) is essential for the function of GrxD (12). Furthermore, evidence shows that GrxD directly interacts with the proteins MiaB, Aft1, Php4, HapX, and BolA to influence redox biology and iron metabolism (13). However, the detailed mechanism through which GrxD controls 2,4-DAPG production remains unclear.

BolA protein is widely distributed in prokaryotes and eukaryotes and has been associated with a range of cellular processes, including biofilm formation, oxidative stress tolerance, bacterial motility, and membrane permeability (14). BolA mediates alterations in bacterial permeability by directly binding to the promoter regions of *dacA*, *dacC*, and *mreB*, which are involved in the regulation of OmpF/OmpC balance (15). Transcriptomic analyses have indicated that BolA recognizes and binds its target genes, which have the 5′-YYGCCAGH-3′ consensus sequence. *Escherichia coli* encodes two proteins of the BolA family, i.e., BolA and IbaG. Notably, BolA and IbaG have different functions: BolA is thought to affect cell shape, whereas IbaG does not (16). IbaG also plays an important role in bacterial growth, cell morphology, and acidic tolerance response. The BolA protein family has been well characterized in various bacterial pathogens, such as *E. coli*, *Klebsiella pneumoniae*, *Salmonella enterica* serovar Typhimurium, and *Vibrio cholerae* (17 – 19), and studies have shown that the BolA protein family regulates bacterial pathogenicity. However, the role of BolA in the plant beneficial of the *P. fluorescens* group remains to be investigated.

Here, we show that the BolA-like protein IbaG plays a critical role in coordinating the expression of plant-beneficial traits in *P. fluorescens* 2P24. We found that IbaG directly interacted with GrxD and controlled 2,4-DAPG production. Moreover, we demonstrated the pleiotropic role of IbaG in regulating cell motility, siderophore production, and acid tolerance. Altogether, our data provide novel insights into the function of IbaG in regulating biocontrol traits of the plant-beneficial bacterium *P. fluorescens* 2P24.

## RESULTS

### Identification of BolA homolog in *P. fluorescens*

**Fig 1:**
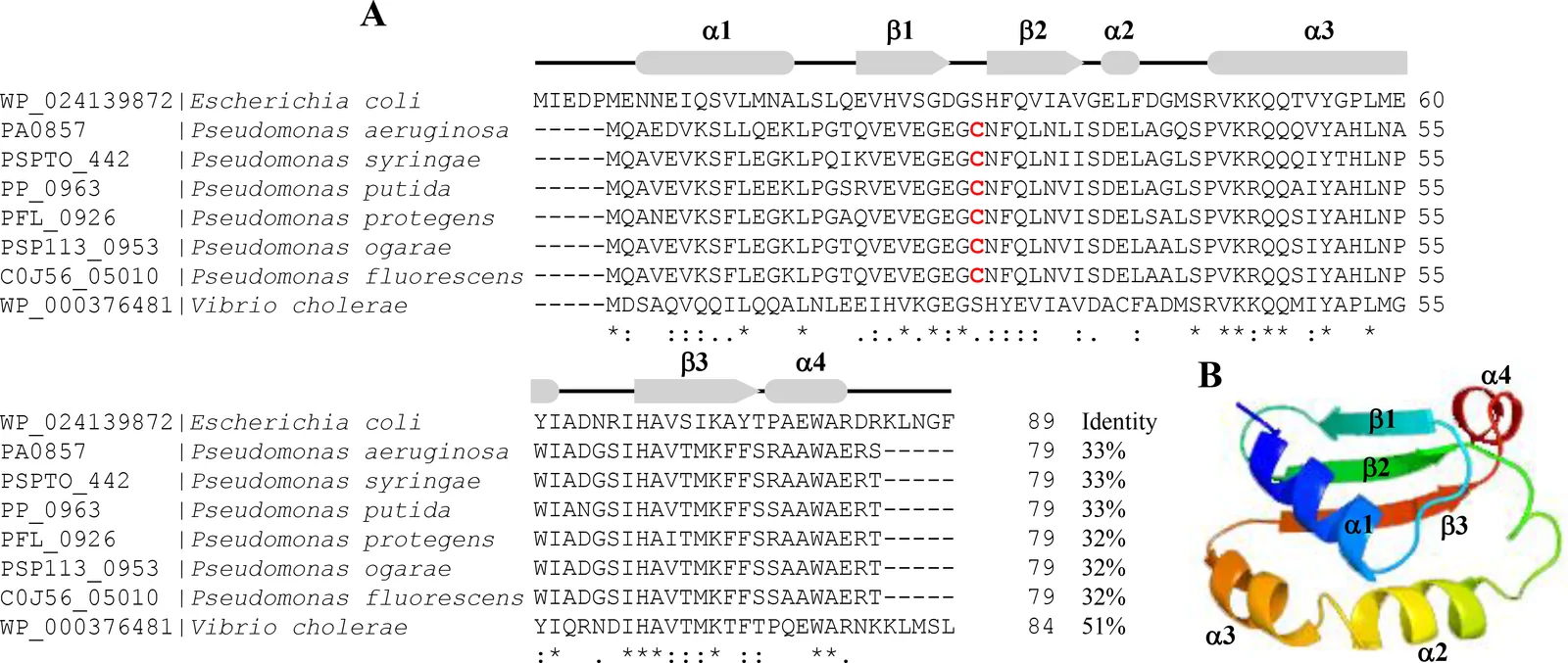
Sequence comparison and secondary structure prediction of BolA-like proteins. (**A**) Clustal omega multiple sequence alignment of *P. fluorescens* IbaG protein with BolA-like proteins from other bacteria. Identical residues (asterisks), conserved residues (colons), and semi-conserved residues (dots) are indicated. The secondary structure of the *P. fluorescens* IbaG protein is shown above the alignment. (**B**) Computer model of the 3D structure predicted for *P. fluorescens* IbaG using the I-TASSER server.

We have previously shown that the monothiol glutaredoxin GrxD positively regulates 2,4-DAPG biosynthesis, and bioinformatic analyses unraveled a potential link between monothiol glutaredoxin and BolA-like proteins (11, 20). To assess the possible role of BolA in regulating the production of 2,4-DAPG mediated by GrxD, we used the amino acid sequence *E. coli* K-12 (GenBank accession no. APC50728.1) to search for BolA homologs in *P. fluorescens* using the psi-BLAST algorithm. We found two proteins homologous to BolA of *E. coli*. Sequence analyses revealed that C0J56_08545 and C0J56_05010 had 42% and 15% sequence identity, respectively, with BolA of *E. coli*. Moreover, they exhibited 26% and 32% sequence identity, respectively, with IbaG of *E. coli*. Thus, we named C0J56_08545 and C0J56_05010 of *P. fluorescens* BolA and IbaG, respectively, based on their homologs in *E. coli*. Structural predications of *P. fluorescens* BolA and IbaG based on *E. coli* BolA protein (PBD ID 2DHM) indicated 100% coverage and 99% confidence (Fig. 1; Fig. S1). The predicted structures of BolA and IbaG had a α1β1β2α2α3β3α4 topology, which is characteristic of the BolA protein family (Fig. 1; Fig. S1).

### IbaG is required for 2,4-DAPG biosynthesis in *P. fluorescens*

**Fig 2:**
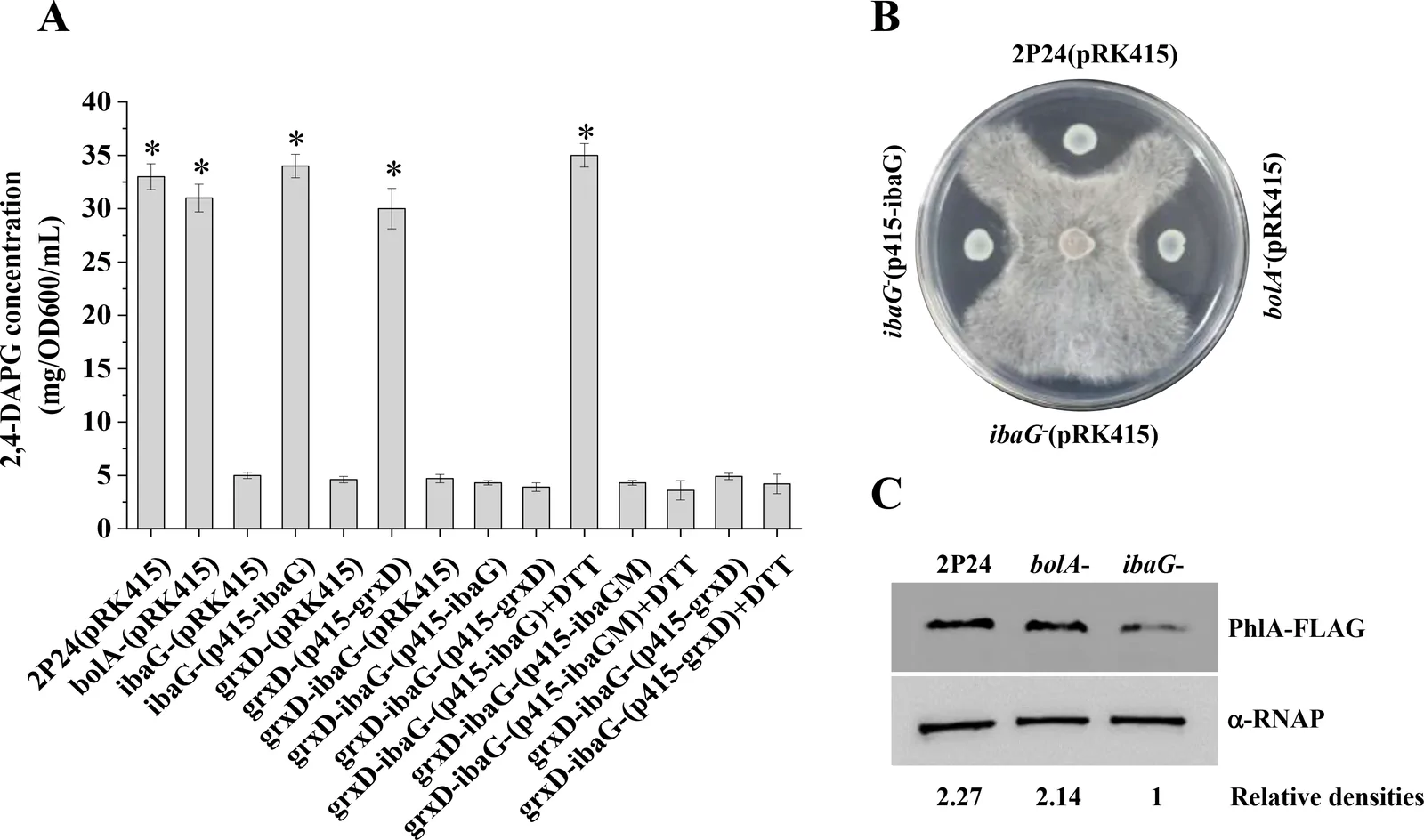
The role of IbaG in the production of 2,4-DAPG in *P. fluorescens*. (**A**) Quantification of 2,4-DAPG by HPLC analysis in strain 2P24 and its derivatives. (**B**) Inhibition of mycelial growth of *Rhizoctoia solani* by 2P24 (pRK415), the *ibaG* mutant (pRK415), the *ibaG* mutant (p415-ibaG), and the *bolA* mutant (pRK415). (**C**) Western blot analysis was performed to detect the levels of PhlA-FLAG in strain 2P24, the *bolA* mutant, and the *ibaG* mutant. The relative intensities of the PhlA-FLAG bands were marked below the bolt. All experiments were performed in triplicate, and * represents significant difference (*P* < 0.05).

To determine whether BolA and IbaG proteins are involved in 2,4-DAPG biosynthesis in *P. fluorescens*, we constructed *bolA* and *ibaG* mutants. High-performance liquid chromatography (HPLC) assays showed that the production of 2,4-DAPG significantly decreased in the *ibaG* mutant compared with that in wild-type *P. fluorescens*. Complementation with the wild-type *ibaG* gene restored 2,4-DAPG production to the wild-type level (Fig. 2A; Fig. S2). In contrast, mutation of the *bolA* gene did not affect 2,4-DAPG biosynthesis (Fig. 2A). Furthermore, the *ibaG* mutant did not inhibit mycelial growth of *R. solani*, whereas the *bolA* mutant retained an inhibitory activity similar to that of wild-type *P. fluorescens* (Fig. 2B). Next, we examined the effects of BolA and IbaG on PhlA protein expression. PhlA protein levels were significantly decreased in the *ibaG* mutant compared with those in the wild-type strain. However, deletion of *bolA* did not affect PhlA protein expression, which was similar to that of the wild-type strain 2P24 (Fig. 2C). In addition, the growth of strain 2P24 and its derivatives was analyzed in KBG broth, and the growth characteristics of the *bolA* and *ibaG* mutants were indistinguishable from those of the wild-type strain (Fig. S3). Altogether, these data suggested that *ibaG*, but not *bolA*, was required for 2,4-DAPG biosynthesis of *P. fluorescens*.

### IbaG interacts with the GrxD

**Fig 3:**
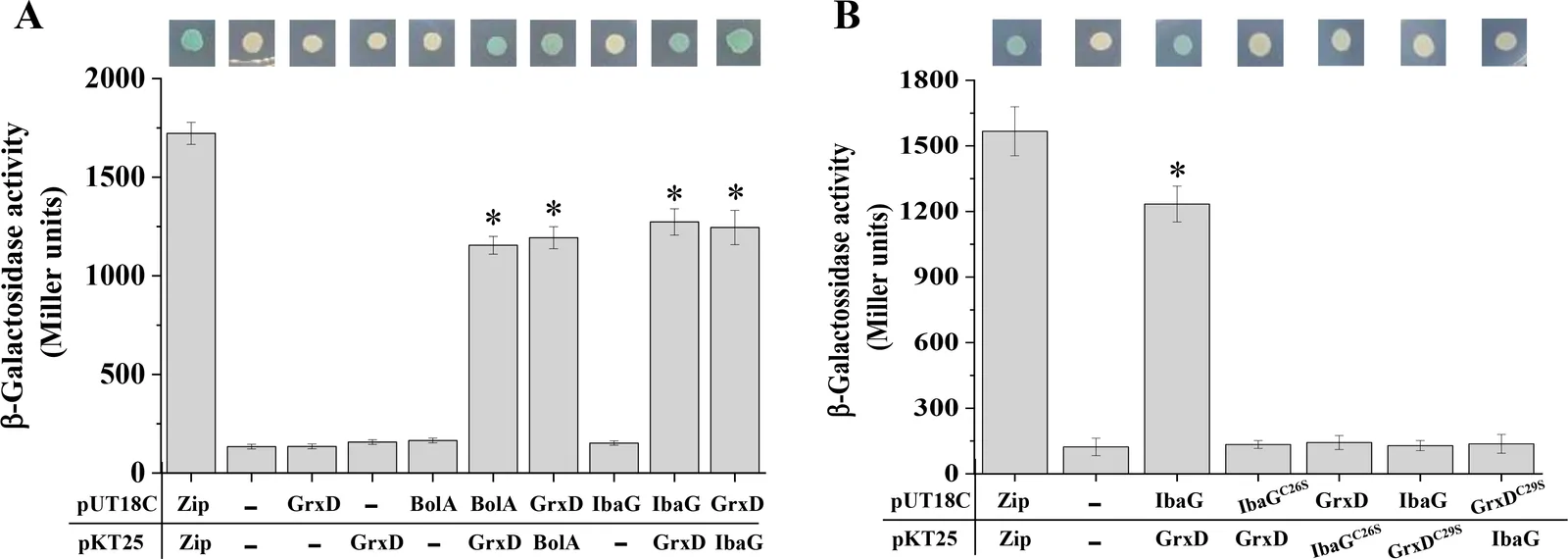
Bacterial adenylate cyclase-based two-hybrid analysis of the interactions between BolA/IbaG and GrxD. (**A**) Quantitative β-galactosidase assays of *E. coli* BTH101 harboring both pKT25- and pUT18C-derived plasmids. Cells were cultured at 30°C for 8 h and β-galactosidase activity was determined as Miller units. (**B**) Mutagenesis of the CGFS motif of GrxD or the conserved C26 of IbaG alters the interactions between IbaG and GrxD. All experiments were performed in triplicate, and * represents significant difference (*P* < 0.05). Zip indicates the positive interaction control.

Grx-BolA interactions occur in the model plant *Arabidopsis thaliana*, and these interactions can influence the redox state of cells (21). To investigate whether IbaG interacted with GrxD in *P. fluorescens*, we used the bacterial adenylate cyclase two-hybrid system. *E. coli* BTH101 co-expressing GrxD and IbaG fusion proteins exhibited significantly higher β-galactosidase activity than the negative control, suggesting an interaction between IbaG and GrxD (Fig. 3A). A similar activity was observed when BolA was used as bait and GrxD as prey (Fig. 3A).

GrxD catalyzes the direct reduction of protein disulfides in a thiol-disulfide exchange reaction (12). Sequence analyses suggested that the C26 residue of IbaG was mostly conserved among different *Pseudomonas* spp. strains (Fig. 1). Therefore, we hypothesized that the C26 residue of IbaG is important for the GrxD-IbaG interaction. To test this hypothesis, we changed the C26 residue to serine and used the mutant version of the protein in the interaction assay. The mutation of the C26 residue of IbaG into serine abolished the interaction between GrxD and IbaG (Fig. 3B). Nuclear magnetic resonance spectroscopy and X-ray crystallography analyses of GrxD (22) have revealed that the CGFS motif is required for the function of GrxD. We found that the CGFS motif of GrxD was necessary for GrxD-IbaG interaction (Fig. 3B). Altogether, our data suggested that the GrxD-IbaG interaction was specific, and the C26 residue of IbaG and the CGFS motif of GrxD were critical for this interaction.

### IbaG is required for GrxD-mediated downregulation of 2,4-DAPG production

Given that GrxD negatively regulates 2,4-DAPG production in *P. fluorescens*, we assessed whether IbaG was involved in this function of GrxD. We constructed a *grxD ibaG* double mutant and measured the production of 2,4-DAPG. The *grxD ibaG* double mutant, as well as the *grxD* mutant or the *ibaG* mutant, produced low levels of 2,4-DAPG (Fig. 2A). We then transformed the *grxD ibaG* double mutant with the wild-type *ibaG* or *grxD* gene and tested whether any of these two genes restored antibiotic production. HPLC assays showed that complementation of the *grxD* mutant with wild-type *grxD* restored 2,4-DAPG production to the wild-type level. Neither *ibaG* nor *grxD* restored 2,4-DAPG production in the *ibaG grxD* double mutant (Fig. 2A). Previous studies have shown that GrxD plays an important role in thiol-disulfide homeostasis. Thus, we hypothesized that GrxD affected the redox state of IbaG, which then regulated 2,4-DAPG production. To test this hypothesis, we cultured bacterial cells in LB medium containing 5 mM DTT (dithiothreitol), which is known to affect the redox state of proteins (12). Expression of IbaG, but not the mutant version IbaG^C26S^, in the *ibaG grxD* double mutant restored the production of 2,4-DAPG to levels observed in strain 2P24 in the presence of DTT (Fig. 2A).

**Fig 4:**
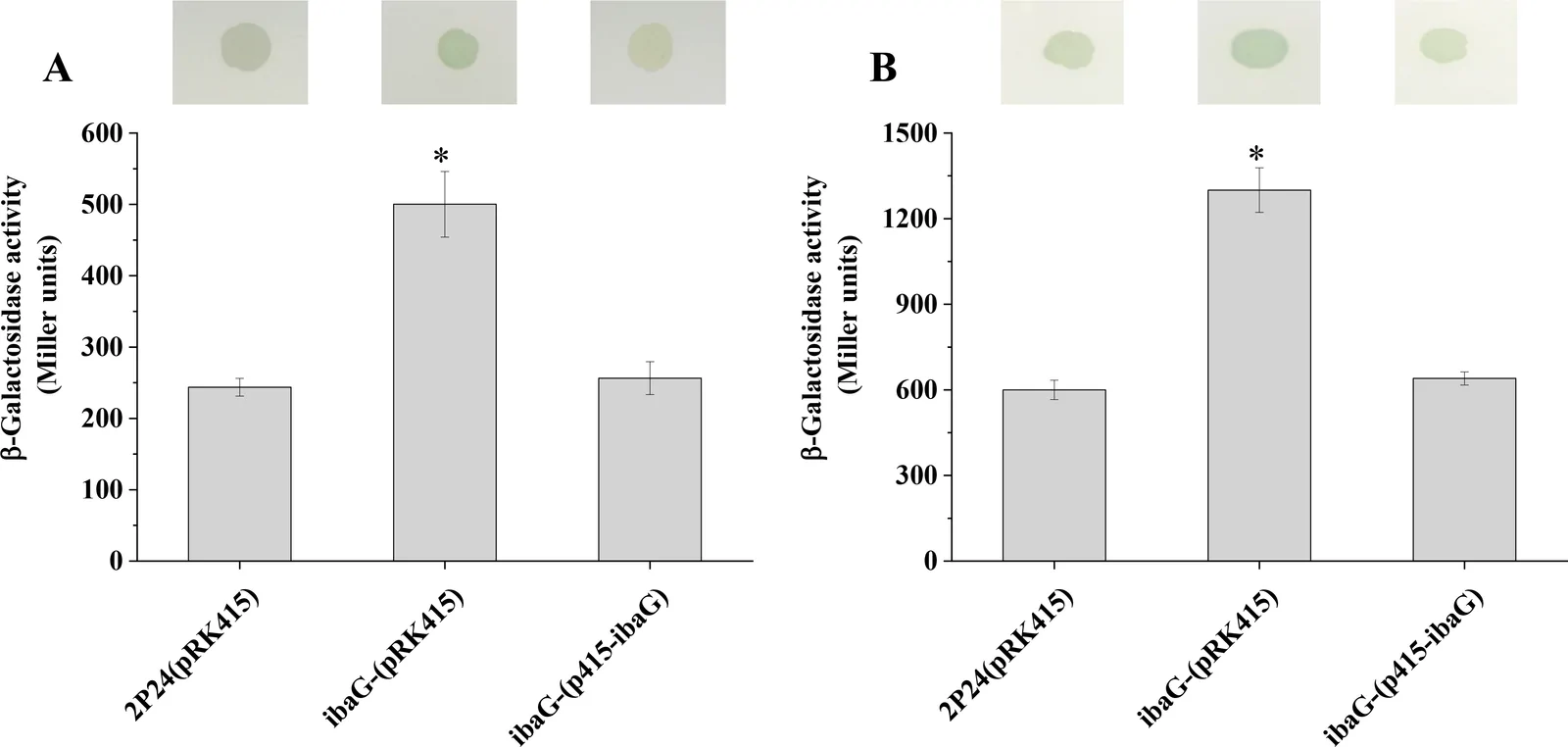
Effect of IbaG on the translational expression of *rsmA* and *rsmE* in *P. fluorescens* 2P24. Expression of *rsmA* (**A**) and *rsmE* (**B**) was measured in 2P24 (pRK415), the *ibaG* mutant (pRK415), and the *ibaG* mutant (p415-ibaG). The strains were inoculated on the LB medium plates supplemented with X-gal. The β-galactosidase activity was determined as Miller units. All experiments were performed in triplicate, and * represents significant difference (*P* < 0.05).

We have previously shown that RsmA and RsmE negatively regulate 2,4-DAPG production (6). Thus, we assessed the effects of *ibaG* on the expression of *rsmA* and *rsmE*. Translational fusion assays and real-time quantitative PCR (RT-qPCR) analyses showed that deletion of *ibaG* significantly increased the expression levels of *rsmA* and *rsmE* (Fig. 4; Fig. S4). These results were consistent with our previous data, showing that GrxD negatively regulates RsmA and RsmE (11). Therefore, IbaG likely regulated 2,4-DAPG production by influencing *rsmA*/*rsmE* expression in strain 2P24. Altogether, these findings showed that IbaG was required for GrxD-mediated downregulation of RsmA and RsmE, and 2,4-DAPG production. Moreover, GrxD likely regulated the function of IbaG by influencing its redox states.

### Identification of IbaG-regulated genes in *P. fluorescens*

**Fig 5:**
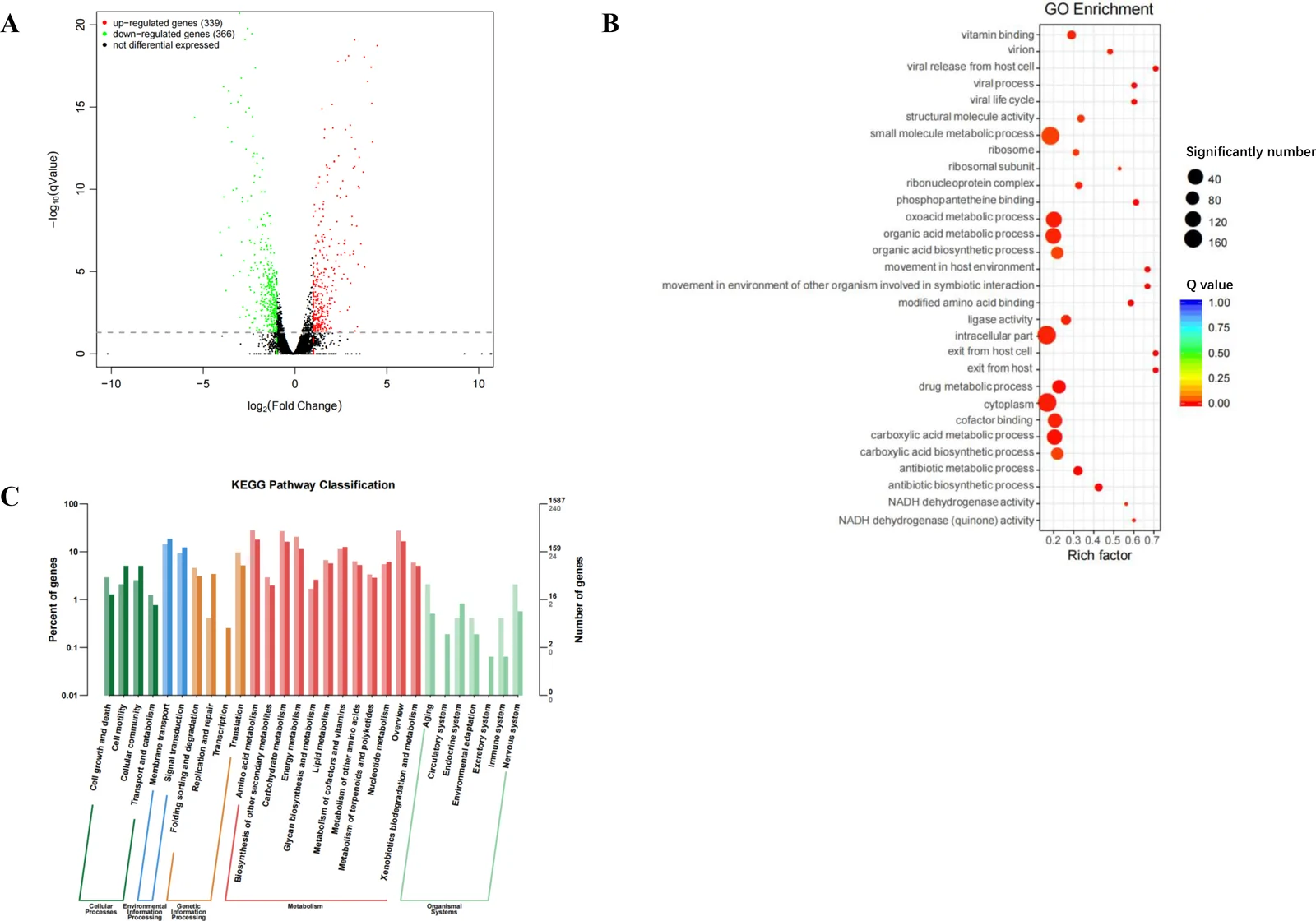
Comparative transcriptomics of the *ibaG* mutant normalized to those of strain 2P24. (**A**) The volcano plot of the DEGs was analyzed in strain 2P24 and its *ibaG* mutant by RNA-seq. Red dots mean up-regulated. Green dots mean down-regulated. (**B**) IbaG regulon categorized by the Gene ontology (GO) functional enrichment analysis. (**C**) Kyoto Encyclopedia of Genes and Genomes (KEGG) enrichment analysis of detected DEGs.

To identify genes, whose expression depended on IbaG, RNA sequencing analyses (RNA-seq) were performed to characterize the transcriptome of strain 2P24 and the *ibaG* mutant. A total of 705 genes were differentially expressed in the *ibaG* mutant compared with the wild-type strain 2P24 (366 genes were downregulated and 339 were upregulated) (Fig. 5). All differentially expressed genes (DEGs) with known functions are summarized in Table S1. The functional classification of the identified genes suggested that the DEGs were mainly involved in fatty acid biosynthesis, amino acid metabolism, and carbon metabolism (Fig. 5B and C). The genes responsible for phage assembly (C0J56_06120, C0J56_06140, C0J56_06105, and C0J56_06165) were all upregulated in the *ibaG* mutant. Consistent with these results, the transcript levels of C0J56_06120, C0J56_06140, C0J56_06105, and C0J56_06165 were significantly higher in the *ibaG* mutant than those in the wild-type strain (Fig. S4). These data suggested that IbaG silenced prophage genes, thus preventing the activation of and lysis by prophages.

The BolA protein family is involved in stress response and bacterial motility. Our transcriptomic results also showed that several stress response-associated genes and genes encoding stress proteins, including C0J56_16570, C0J56_24075, and C0J56_19920, were significantly downregulated in the *ibaG* mutant. Genes responsible for flagellar assembly (FlgF and FlgC) and the chemotaxis response regulator CheY were also downregulated in the *ibaG* mutant. Additionally, the *ibaG* regulon includes genes involved in Fe-S cluster biosynthesis and assembly, secretion system, amino acid metabolism, transporter, signal transduction, and other functions.

To verify the RNA-seq data, we randomly selected four genes (*cheY*, *iscR*, *gabP*, and *purU*) from the identified DEGs. Transcriptional fusion assays indicated that the expression of *cheY* and *iscR* significantly decreased, whereas that of *gabP* and *purU* significantly increased in the *ibaG* mutant compared with that in the wild-type strain, thus validating the RNA-seq data (Fig. S5).

Altogether, our transcriptome analysis suggested that IbaG plays a global role in transcriptional regulation and can influence different cellular processes, including Fe-S cluster biosynthesis, flagella assembly, motility, and stress response.

### Effect of IbaG on acid resistance and siderophore production in *P. fluorescens*

**Fig 6:**
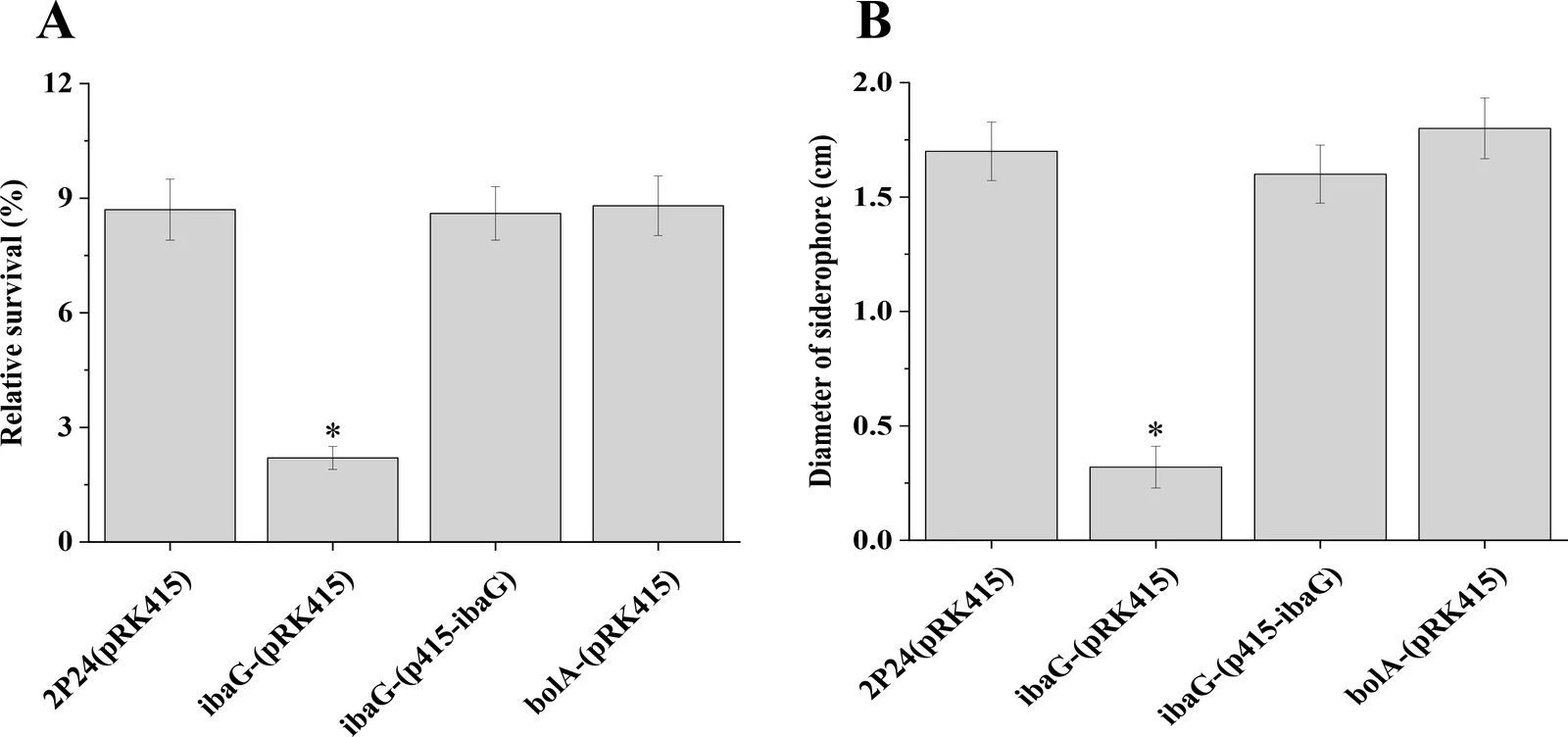
Effect of IbaG on the acid tolerance and siderophore production of *P. fluorescens*. (**A**) Acid tolerance was determined by calculating the proportion of cells that survived during growth in acidic conditions (LB medium at pH 5.5) versus in normal conditions (LB medium at pH 7.0). (**B**) The production of siderophore of strain 2P24 and its derivatives was determined by measuring the diameter of the orange circle on the chrome azurol S (CAS) plates. The experiments were performed in triplicate, and * represents significant difference (*P* < 0.05).

Given that IbaG of *E. coli* influences acid tolerance (16), we explored whether the absence of *ibaG* impaired *P. fluorescens* survival following exposure to acidic stress. Our data showed that the survival of the *ibaG* mutant was significantly lower than that of the wild-type and complemented strains (Fig. 6A). Notably, the mutation of *bolA* did not affect survival under an acid stress condition (Fig. 6A). These data suggested the important role of *ibaG* in growth under acidic conditions.

Siderophore production by strain 2P24 is partially responsible for promoting plant growth (9). A previous study has shown the downregulation of siderophore production in the *grxD* mutant (11). Therefore, we compared siderophore production in the wild-type and *ibaG* mutant strains and found that the *ibaG* mutant produced less siderophore than the wild-type and *bolA* mutant strains (Fig. 6B). This result is consistent with the RNA-seq data that showed that the levels of *pvdP*, *pvdE*, and C0J56_20220, which encoded proteins involved in the synthesis of a primary siderophore, significantly decreased in the *ibaG* mutant (Table S1). We further analyzed the effects of IbaG on *pvdP*, *pvdE*, and C0J56_20220 expression using RT-qPCR and confirmed a significant induction of these genes (Fig. S4). Collectively, these results demonstrate that IbaG is involved in siderophore production in *P. fluorescens*.

### IbaG is required for swimming motility and root colonization of *P. fluorescens*

**Fig 7:**
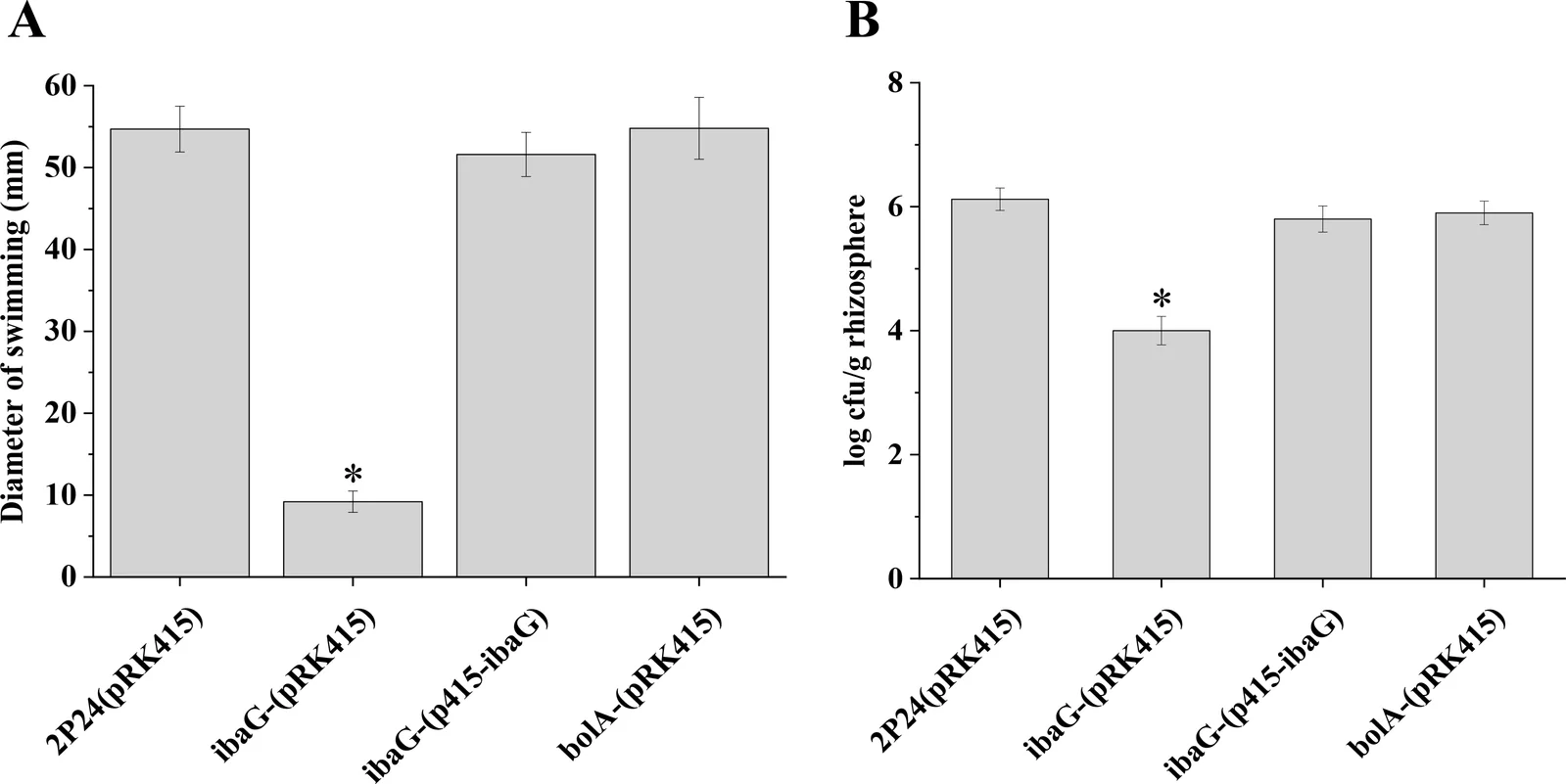
Swimming motility and colonization of wheat rhizosphere by *P. fluorescens* 2P24 and its derivatives. (**A**) The colony diameter of strain 2P24 and its derivatives was measured in swimming assay. (**B**) The rhizosphere population of strain 2P24 (pRK415), the *ibaG* mutant (pRK415), the *ibaG* mutant (p415-ibaG), and the *bolA* mutant (pRK415) were determined at 14 day after inoculation. All experiments were performed in triplicate, and * represents significant difference (*P* < 0.05).

Motility is a key bacterial trait for root colonization and is probably involved in biocontrol activities. We compared the swimming motility of strain 2P24 and *ibaG* mutant. The swimming motility of the *ibaG* mutant was significantly reduced compared with that of the wild-type strain (Fig. 7A). Complementation of the *ibaG* mutant with p415-ibaG restored the swimming motility, suggesting that *ibaG* positively regulated swimming motility. However, the mutation of *bolA* did not influence bacterial swimming motility (Fig. 7A). We then investigated whether the loss of swimming motility in the *ibaG* mutant was due to the influence of the growth or cellular morphology of strain 2P24 and found no significant difference between the *ibaG* mutant and wild-type strain (Fig. S3).

To determine the contribution of IbaG to the root colonization of *P. fluorescens* 2P24, we conducted wheat root colonization assays under greenhouse conditions. Strain 2P24 and its derivatives were introduced into sterile soil. The ability of the *ibaG* mutant to colonize the root rhizosphere was significantly impaired 14 days after inoculation compared with that of the wild-type and *bolA* mutants (Fig. 7B). Thus, the *ibaG* gene is important for rhizosphere colonization of *P. fluorescens*.

## DISCUSSION

*P. fluorescens* 2P24 is an effective biocontrol agent that can prevent several soilborne diseases by directly inhibiting the pathogen’s growth through the production of 2,4-DAPG (9). Rhizosphere colonization is required for strain 2P24 to protect plants from phytopathogens. Colonization by strain 2P24 relies on a complicated interaction between the plant, pathogens, and environmental stimuli (23). We recently reported that GrxD is required for the production of 2,4-DAPG and rhizosphere colonization by strain 2P24 (11), although the underlying molecular mechanism remains largely elusive. Here, we report that GrxD regulated the production of 2,4-DAPG likely by modulating the activity of the BolA family protein IbaG in *P. fluorescens* 2P24. This conclusion is supported by the following results: (i) the *ibaG* mutant, like the *grxD* and *ibaG grxD* double mutants, produced undetectable amounts of 2,4-DAPG; (ii) GrxD protein directly interacted with IbaG; (iii) the C26 of IbaG and the C29 of GrxD were needed for IbaG-GrxD interaction; (iv) expression of a functional IbaG, but not GrxD, restored 2,4-DAPG production in the *ibaG grxD* double mutant; (v) DTT, which mimics the function of GrxD, was needed for the function of IbaG. DTT is a known strong reducing agent that can block the formation of disulfide bonds. A previous study has shown that the redox shift pattern of At3g11630 of *Arabidopsis thaliana* changes in the presence of DTT (24). In *Vibrio cholerae*, TcpP forms a dimer through an intermolecular disulfide bond, and the dimer formation is abolished with 10 mM DTT (25). In *Saccharomyces cerevisiae*, cytosolic Grx3/4 influences the maturation of Fe-S clusters by interfering with the function of the BolA-like proteins Fra1 and Fra2, which regulate the expression of the iron transport system (26). Moreover, the function of the transcriptional regulator OxyR is regulated by Grx1 through the formation of a disulfide bond in *E. coli* (27). Therefore, GrxD directly interacts with IbaG and influences the redox state of IbaG which regulates the production of 2,4-DAPG. BolA-like proteins have been extensively studied in prokaryotes and eukaryotes and were shown to play important roles in different bacterial physiological processes, including virulence-related traits. To our knowledge, the present study is the first to report that a BolA family protein, specifically IbaG of *P. fluorescens* 2P24, regulates antibiotic production of plant-associated beneficial bacteria.

We investigated further the mechanism underlying the regulation of 2,4-DAPG production by IbaG. We found that mutation of *ibaG* increased the translation of RsmA and RsmE but decreased the levels of the 2,4-DAPG biosynthesis-associated gene *phlA* (Fig. 2C). These results are consistent with a previous report showing that RsmA/E directly bind to the 5′ end untranslated region of *phlA* mRNA to block its translation (6). Overall, these data suggest that GrxD activates IbaG, which downregulates the expression of RsmA/E, consequently promoting the translation of genes involved in 2,4-DAPG biosynthesis and activating the 2,4-DAPG production. Phosphorylation of BolA is critical for the function of BolA in *E. coli*. Specifically, four phosphorylation sites (S26, S45, T81, and S95) are responsible for the stability of BolA protein (28). Future work is needed to explore whether post-translational modifications of IbaG in *P. flurescens* 2P24 are a regulating IbaG control of the expression of its target genes.

Although there is a significant homology among BolA-like proteins, our results showed that the functions of IbaG and BolA in regulating biocontrol-related traits of *P. fluorescens* differ. Protein-protein interaction assays showed that BolA and IbaG interacted with GrxD (Fig. 3), whereas IbaG, but not BolA, was involved in the regulation of biocontrol-related traits of strain 2P24. For example, mutation of *ibaG* reduced the production of 2,4-DAPG, which was not different between the *bolA* mutant and wild-type strains (Fig. 1A). Moreover, siderophore production, bacterial swimming motility, and acid tolerance were significantly decreased in the *ibaG* mutant but not in the *bolA* mutant (Fig. 6 and 7). Transcriptome data suggested that IbaG direct effects were related to the regulation of the expression of flagellar and stress response-associated genes (Table S1). Additionally, inducing the expression of BolA from a plasmid failed to complement the phenotype of the *ibaG* mutant (data not shown), suggesting that IbaG and BolA proteins have different functions in *P. fluorescens*. Such functional differences among the BolA-like proteins have previously been reported in *E. coli*, *V. cholerae*, and *Sinorhizobium meliloti*, indicating that BolA and IbaG may be involved in different regulatory pathways to allow adaptation to the constantly changing environment (16, 18, 29).

A previous study showed higher cellular levels of cyclic-di-GMP (c-di-GMP) in an *E. coli bolA* mutant (19). The secondary messenger c-di-GMP plays a pleiotropic role in many biological processes. In strain 2P24, c-di-GMP was reported to regulate the biocontrol traits, including 2,4-DAPG production, swimming motility, and biofilm formation (30). However, our transcriptome data did not reveal any difference in the expression of genes involved in c-di-GMP metabolism between the *ibaG* mutant and wild-type strain, suggesting that IbaG modulated 2,4-DAPG biosynthesis independently from the c-di-GMP regulatory pathway (Table S1).

Overall, our results demonstrated that the *ibaG* gene is involved in 2,4-DAPG production mediated by GrxD, the swimming motility, and the acid tolerance of *P. fluorescens*. This finding highlights the complexity of the regulation of 2,4-DAPG production and provides new insights for fine-tuning the biocontrol traits of *P. fluorescens* under adverse environmental conditions.

## MATERIALS AND METHODS

### Bacterial strains and culture conditions

The strains and plasmids used in this study are listed in Table 1. *P. fluorescens* 2P24 and *E. coli* DH5α strains were routinely grown in lysogeny broth (LB), KB, or ABM medium at 28°C. Antibiotics were supplemented when required for plasmid maintenance or transformation at the following concentrations: ampicillin (50 µg/mL), kanamycin (50 µg/mL), and tetracycline (20 µg/mL).

**TABLE 1:** Bacterial strains and plasmids used in this study

Strains, plasmids, or oligonucleotides	Relevant characteristics^*a*^	Reference or source
Strains		
*P. fluorescens*		
2P24	Wild type, Ap^r^	(23)
WPM31	In-frame deletion of *grxD*, Ap^r^	(11)
WPM81	In-frame deletion of *ibaG*, Ap^r^	This work
WPM82	In-frame deletion of *bolA*, Ap^r^	This work
WPM83	Double deletion of *ibaG* and *grxD*, Ap^r^	This work
WPM28	Strain 2P24 with a FLAG epitope sequence tagged to the C terminus of PhlA, Ap^r^	(6)
WPM84	WPM81 with a FLAG epitope sequence tagged to the C terminus of PhlA, Ap^r^	This work
WPM85	WPM82 with a FLAG epitope sequence tagged to the C terminus of PhlA, Ap^r^	This work
*Rhizoctonia solani*	Basidiomycete fungus caused cotton blight	Lab stock
*E. coli*		
DH5α	*supE44 lacU*169 (*ϕ80lacZ* M15) *hsdR*17 *recA*1 *endA*1 *gyrA*96 *thi*-1 *relA*1	(31)
BTH101	*F-, cya-99, araD139, galE15, galK16, rpsL1 (Strr), hsdR2, mcrA1, mcrB1*	Euromedex
Plasmids		
p2P24Km	Sucrose-based counter-selectable plasmid, Km^r^	(6)
p2P24Km-ibaG	Plasmid p2P24Km carrying a deleted *ibaG* gene, Km^r^	This work
p2P24Km-bolA	Plasmid p2P24Km carrying a deleted *bolA* gene, Km^r^	This work
pKT25	pSU40 derivative with T25 fragment of CyaA, Km^r^	Euromedex
pUT18C	pUC19 derivative with T18 fragment of CyaA, C-terminal fusions, Ap^r^	Euromedex
pKT25-grxD	pKT25 containing the *grxD* gene, Km^r^	This work
pUT18C-ibaG	pUT18C containing the *ibaG* gene, Ap^r^	This work
pKT25-ibaG	pKT25 containing the *ibaG* gene, Km^r^	This work
pUT18C-grxD	pUT18C containing the *grxD* gene, Ap^r^	This work
pKT25-grxDC29S	pKT25 containing *grxD* ^C29S^, Ap^r^	This work
pUT18C-ibaGC26S	pKT25 containing *ibaG* ^C26S^, Ap^r^	This work
pUT18C-grxDC29S	pKT25 containing *grxD* ^C29S^, Ap^r^	This work
pKT25-ibaGC26S	pKT25 containing *ibaG* ^C26S^, Ap^r^	This work
pKT25-bolA	pKT25 containing the *bolA* gene, Km^r^	This work
pUT18C-bolA	pUT18C containing the *bolA* gene, Ap^r^	This work
pKT25-ZIP	pKT25 derivative with the leucine zipper of GCN4, Km^r^	Euromedex
PUT18C-ZIP	pUT18C derivative with the leucine zipper of GCN4, Ap^r^	Euromedex
p6013-rsmA	*rsmA*′-′*lacZ* translational fusion, Tet^r^	(6)
p6013-rsmE	*rsmE*′-′*lacZ* translational fusion, Tet^r^	(6)
pRK415	Broad-host-range cloning vector, Tet^r^	(32)
p415-grxD	pRK415 containing the *grxD* gene, Tet^r^	(11)
p415-ibaG	pRK415 containing the *ibaG* gene, Tet^r^	This work
p415-ibaGM	pRK415 containing *ibaG* ^C26S^, Tet^r^	This work

^*a*^ Ap, ampicillin; Km, kanamycin; Tet, tetracycline.

### Gene deletion in *P. fluorescens*

The markerless *P. fluorescens ibaG* deletion mutant was constructed as follows. The flanking regions of the *ibaG* gene were amplified by PCR (primers are listed in Table 2). The PCR products of the upstream and downstream regions were gel extracted and then connected by fusion PCR to delete *ibaG*. The fusion PCR product was digested with EcoRI and BamHI and then cloned into the suicide plasmid p2P24Km (6) (Fig. S6), yielding p2P24-ibaG. Plasmid p2P24-ibaG was transferred into *P. fluorescens* 2P24 by electroporation. Plasmid p2P24-ibaG was integrated into the chromosome of strain 2P24 by the first crossover and selected on LB plates with kanamycin. Cells with the second crossover to generate the deletion were selected by culture on LB plates containing 5% sucrose. The *ibaG* mutant was confirmed by amplified and Sanger sequencing. In the same way, the in-frame knockout mutant of *bolA* was constructed using the primers listed in Table 2.

**TABLE 2:** Primers used in this study

Name	Sequence (5′−3′)^*a*^	Comment
bolA-HindIII-F1	GGCAAGCTTCGGGGTTGAGGCCCGGCGTTTTTTAG	Constructing an in-frame *bolA* deletion mutant
bolA-R1	TCAGTGTTTGCTGCCACCGGCACATTCGATGCGTTGTTGCATGCTCAT	
bolA-F2	ATGAGCATGCAACAACGCATCGAATGTGCCGGTGGCAGCAAACACTGA	
bolA-EcoRI-R2	GGGAATTCGCGAAAGCTGGTGGTCTTGGGGTC	
ibaG-EcoRI-F1	ATGAATTCAAGCAGGACCCAAGCGCGTTCTATG	Constructing an in-frame *ibaG* deletion mutant
ibaG-R1	CTTGGGCTCAGGTGCGCTCGGCCCATACAGCCTGCATGCTCAACCTCAAT	
ibaG-F2	ATTGAGGTTGAGCATGCAGGCTGTATGGGCCGAGCGCACCTGAGCCCAAG	
ibaG-BamHI-R2	ATGGATCCGCTTGCCGTGCATGTTCACTTCG	
pKT25-grxD-F	GGGTCGACTGTGGATATCATCGAAACGAT	Cloning the coding region of *grxD* into pKT25
pKT25-grxD-R	GGGGATCCTCGGCTTCGGCCTTGTTCGCCG	
pUT18C-bolA-SalI	AGGTCGACTATGAGCATGCAACAACGCATC	Cloning the coding region of *bolA* into pUT18C
pUT18C-bolA-BamHI	GGGGATCCTCTCAGTGTTTGCTGCCACCGGCAC	
pKT25-bolA-F	ACTCTAGAGATGCTGATTCTGACCC	Cloning the coding region of *bolA* into pKT25
pKT25-bolA-R	TCGAATTCATAATGGCTTGGTTCGA	
pUT18C-ibaG-PstI	ACACTGCAGGATGCAGGCTGTAGAAGTGAAGAG	Cloning the coding region of *ibaG* into pUT18C
pUT18C-ibaG-EcoRI	ATGAATTCGATCAGGTGCGCTCGGCCCAGG	
pKT25-ibaG-F	CGGGTACCTGGTGCGCTCGGCCCA	Cloning the coding region of *ibaG* into pKT25
pKT25-ibaG-R	ACTCTAGAGATGCAGGCTGTAGAAGTG	
ibaG-C26S-F	GTTGAAGGCGAAGGCGCCAACTTCCAGTT	Site-directed mutagenesis of C26 to S of IbaG
ibaG-C26S-R	GCGCCTTCGCCTTCAACTTCCACCTGCGT	

^*a*^ Restriction sites inserted in the primer for the cloning are underlined.

For the complementation of *ibaG*, the *ibaG* gene and its native promoter region were amplified by PCR using 2P24 genomic DNA as a template (primers are listed in Table 2). The PCR product was then ligated into the shuttle plasmid pRK415 (32), yielding p415-ibaG. The complementation plasmid p415-ibaG was introduced into the corresponding *ibaG* mutant by electroporation.

### Bioinformatic analyses

Amino acid alignment of BolA or IbaG homologs was performed using the Clustal Omega multiple sequence alignment tool at the European Bioinformatics Institute (EMBL-EBI) website (https://www.ebi.ac.uk/Tools/msa/clustalo/). Protein structural prediction was performed using the I-TASSER server (https://zhanggroup.org/I-TASSER/).

### Site-directed mutagenesis

The site-directed point mutant of *ibaG* was performed by using the QuikChange site-directed mutagenesis kit according to the manufacturer’s protocol (Agilent Technologies, USA). Primers are listed in Table 2. Substitutions were confirmed by Sanger sequencing.

### Immunoblot analyses

Strain 2P24, the *bolA* mutant, and the *ibaG* mutant were grown overnight on LB agar and then cultured in liquid LB medium to an OD600 of 1.0 (ca. 1 × 10^8^ CFU/mL) (Fig. S7) and 1 mL samples were taken. Cells were collected by centrifugation, resuspended in 1× phosphate-buffered saline (PBS) buffer, and lysed by sonication. Samples were separated on a 10% SDS-PAGE gel and blotted on a polyvinylidene difluoride membrane according to the manufacturer’s instructions (Bio-Rad, USA). The blotting membrane was blocked in blocking buffer (5% milk powder) for 1 h at room temperature and then incubated with an anti-FLAG antibody (Sangon Biotech, Shanghai, China). After washing with Tris-buffered saline with Tween 20 buffer, the membrane was then incubated with horseradish peroxidase-goat anti-mouse IgG secondary antibody, and the signal was detected using a chemiluminescence detection kit (Thermo Fisher, USA). RNA polymerase beta was used as the reference protein as the internal standard (Thermo Fisher, USA).

### Bacterial two-hybrid assays

Bacterial two-hybrid assays were performed as described previously (33). The *grxD* and *ibaG* genes were amplified and cloned into the pUC18C and pKT25 vectors in frame with two gene fragments of the adenylate cyclase of *Bordetella pertussis*. The resulting vectors were then cotransformed into *E. coli* BTH101 and protein interactions were assessed by β-galactosidase activity (34).

### Quantification of 2,4-DAPG

Strain 2P24 and its derivatives were grown in 30 mL KBG (KB broth with 2% glucose) at 30°C for 36 h. The fermentation culture was mixed with 0.1% (vol/vol) formic acid and then extracted with an equal volume of ethyl acetate as previously described (35). The ethyl acetate extract was dried and resuspended in 50 µL of methanol. A 10-µL aliquot of the extracted sample was then detected by HPLC analysis (Waters 2489) using the following conditions: C18 reversed-phase column (5 µm, 4.5 × 250 mm, Agilent) eluted with 10% acetonitrile (vol/vol). Quantification of 2,4-DAPG was performed by integrating the area under the curve at 300 nm and compared with the standard curve prepared by injection of purified 2,4-DAPG.

### RNA-seq analysis

Cells of strain 2P24 and its *ibaG* mutant were cultured to the exponential phase (OD600 = 1.0) in LB medium. Three biological replicates were prepared for each strain. The cells (10 mL) were collected and centrifuged for 3 min at 9,600 × *g*. The total RNA was extracted using the RNeasy minikit (Qiagen, MD, USA). Extracted RNA was treated with Turbo DNase I (Thermo Fisher, USA) to remove genomic DNA contaminants. The rRNA was removed with the Ribo-Zero rRNA removal Kit (Illumina, USA). The integrity of the total RNA was then assessed with the Agilent TapeStation System (Agilent Technologies, UK). The cDNA library was constructed using the NEBNext UltraTM II RNA Library Prep Kit, which was then sequenced using Illumina HiSeq 4000 in rapid mode at a read length of 100 bp paired ends by Sangon Biotech Co., Ltd. (Shanghai, China). The RNA-seq raw data were mapped to the genome of *P. fluorescens* 2P24 (GenBank accession no. CP025542) using HISAT software. The DESeq2 method was used to identify the DEGs of the *ibaG* mutant compared to the wild type (36), and a log2 fold change of ≥1 and a *P* value (adjusted) of <0.05 were used to establish significance. The volcano plots were generated using the R language *ggplots* 2 package. To identify the potential pathways that the DEGs were involved, KEGG pathway enrichment analysis was performed using DAVID (https://david.cifcrf.gov/). The GO analysis was performed with Blast2GO (BioBam) (http://bowtie-bio.sourceforge.net/index.shtml).

### Real-time quantitative PCR assay

The validation of the RNA-seq analysis was performed by RT-qPCR. After RNA isolation and DNase I treatment, reverse transcription was done with cDNA Synthesis SuperMix (TransGen Biotech, Beijing, China). RT-qPCR was carried out with a TaqMan PCR master mix (Thermo Fisher, USA). Normalization was performed against the 16S rRNA gene using the 2^−ΔΔCt^ method.

### Siderophore assay

Siderophore production was determined by CAS assay (37). Briefly, strain 2P24 and its derivatives grown overnight were diluted to an OD600 of 0.6, and 5 mL of each culture was dropped onto CAS plates. Plates were incubated at 30°C for 36 h, and the production of siderophore was determined by measuring the diameter of an orange zone.

### Swimming motility assays

Swimming assays were performed as previously described (11). Briefly, 2P24 and its derivatives were cultured overnight. Five-microliter aliquots of culture were then spotted onto the center of LB plate with 0.5% agar. The plates were incubated for 16 h at 30°C. The zone of swimming motility was recorded by measuring the diameter of the halo.

### Rhizosphere colonization

Rhizosphere colonization was performed as described previously (24). Briefly, strain 2P24 and its derivatives were labeled by streptomycin sulfate resistance (38). Surface-sterilized wheat seeds (*Triticum aestivum* cultivar Yumai 49) were soaked inside the bacterial cultures (10^8^ CFU/mL) before sowing into seedling plates containing sterile soil. After 10 days of growth, ten plants were harvested randomly from each treatment, and bacteria were recovered from the rhizosphere by vortexing the root tips (last centimeter of the main root) for 2 min in a tube containing 5 mL of PBS buffer. The samples were serially diluted and then plated on LB agar plates. The experiment was performed three times with three replicates, and population data, collected as CFU counts, were log_10_ transformed before statistical analysis.

### Statistical analysis

Statistical analysis was performed by the least significant difference test or the Student’s *t* test. Data were presented as mean values ± standard deviations, and *P* values less than 0.05 were considered statistically significant. All experiments were performed at least three times independently to confirm reproducibility.

## Data Availability

The raw data of RNA-sequencing were submitted to the Short Read Archive (SRA) at the NCBI database under accession number PRJNA956275.

## SUPPLEMENTAL MATERIAL

Supplemental figures spectrum.00985-23-s0001.doc Fig. S1 to S7.

Table S1 spectrum.00985-23-s0002.xlsx Results of RNA-seq analysis. List of 705 genes with altered expression identified by comparison of the transcriptomes of the ibaG mutant and the wild type [a fold change of {greater than or equal to} 1 and a *P* value (adjusted) of < 0.05 were used to establish significance].

ASM does not own the copyrights to Supplemental Material that may be linked to, or accessed through, an article. The authors have granted ASM a non-exclusive, world-wide license to publish the Supplemental Material files. Please contact the corresponding author directly for reuse.
