# Supplementary material for: Regulation of 2,4-diacetylphloroglucinol biosynthesis and biocontrol capacity by the BolA family protein IbaG in Pseudomonas fluorescens 2P24
Source: Microbiol Spectr. 2023 Sep 19;11(5):e00985-23. doi: 10.1128/spectrum.00985-23 (PMC10580914; doi:10.1128/spectrum.00985-23)
Supplement: Supplemental figures — Fig. S1 to S7. [file spectrum.00985-23-s0001.doc]

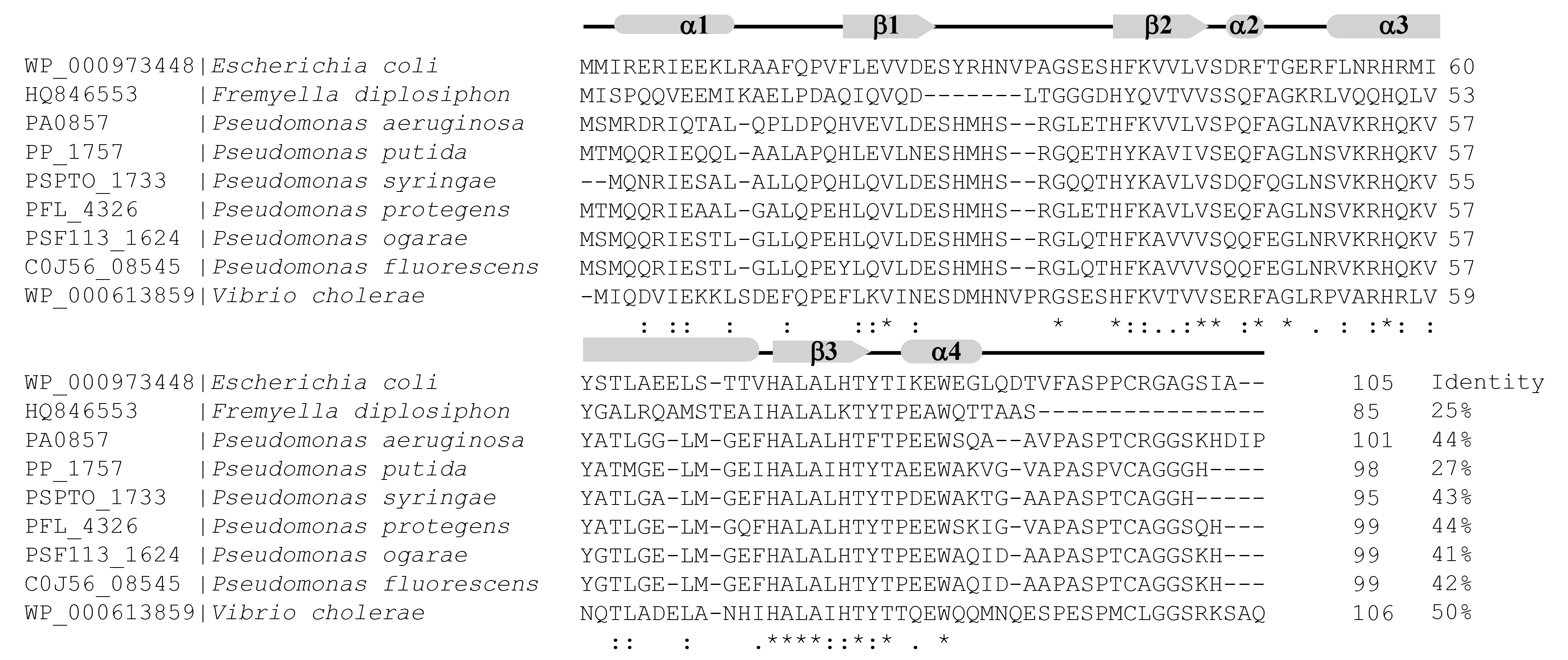
**Supporting information**

Figure S1. Clustal omega multiple sequence alignment of *P. fluorescens* BolA protein with BolA-like proteins from other bacteria. Identical residues (asterisks), conserved residues (colons), and semi-conserved residues (dots) are indicated. The secondary structure regarding *P. fluorescens* BolA protein is shown above the alignment.


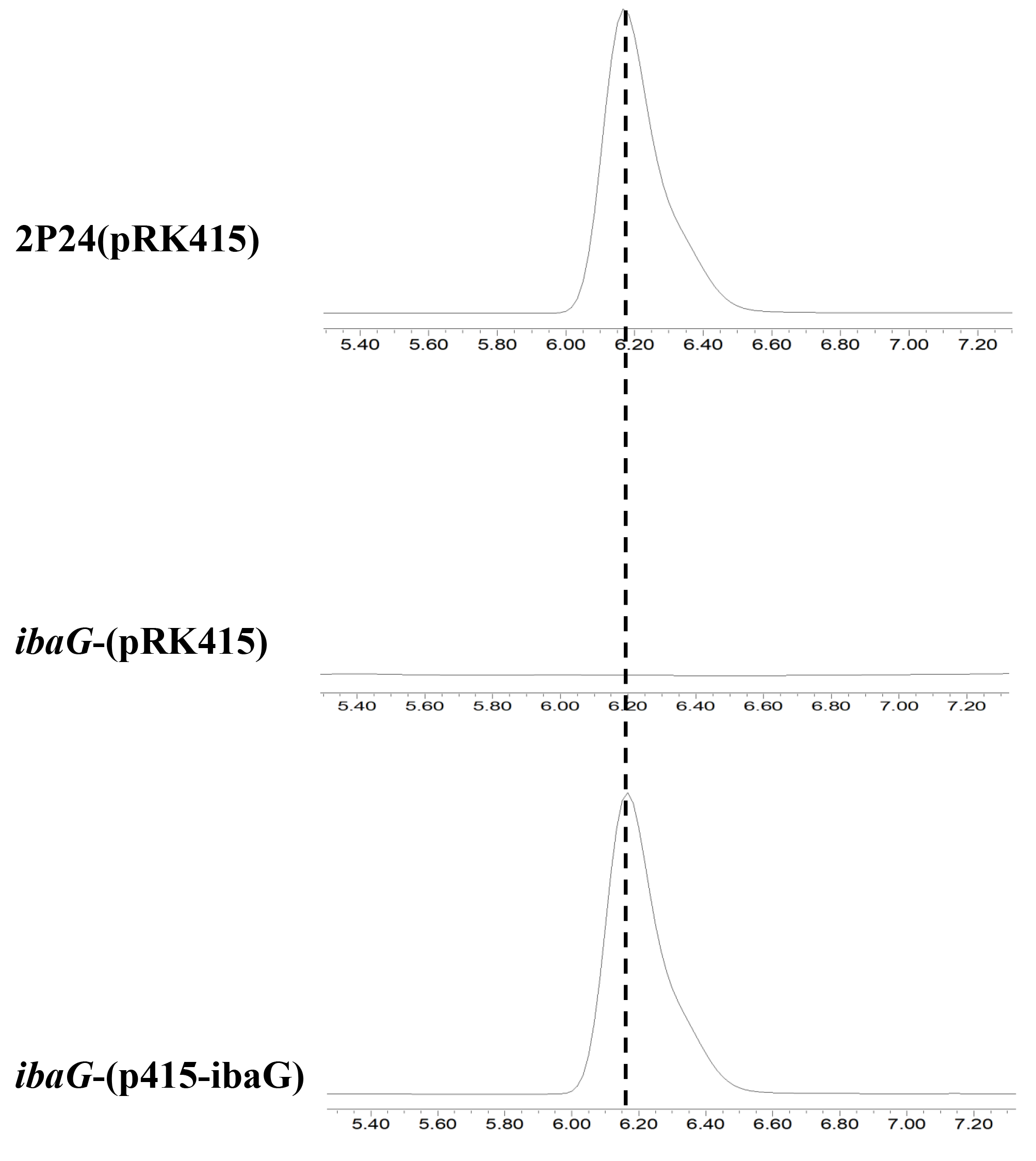


Figure S2. HPLC profiles for detecting the 2,4-DAPG production of the *ibaG* mutant and its complemented strain compared with wild-type strain 2P24.


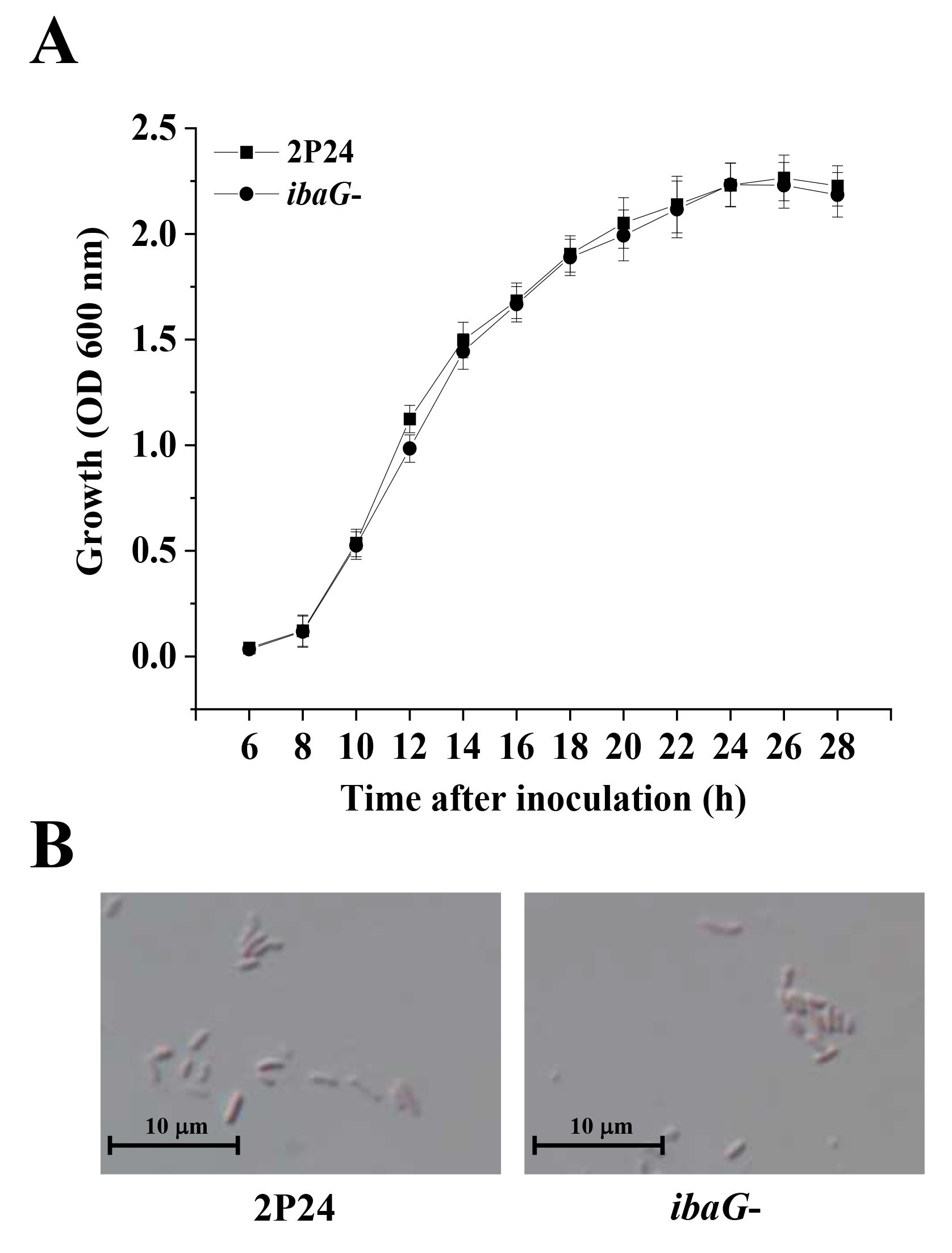


Figure S3. The effect of *ibaG* gene on the growth and cell morphology of *Pseudomonas fluorescens*. (A) Wild type strain 2P24 and the *ibaG* mutant were cultured in KBG medium and the absorbance at 600 nm were checked at different time points of the growth curve. The experiments were performed in triplicate and the mean values ± standard deviations are indicated. (B) Cells of the wild type 2P24 and its *ibaG* mutant were stained by Gram staining assay, and the images under a light microscope were then photographed.


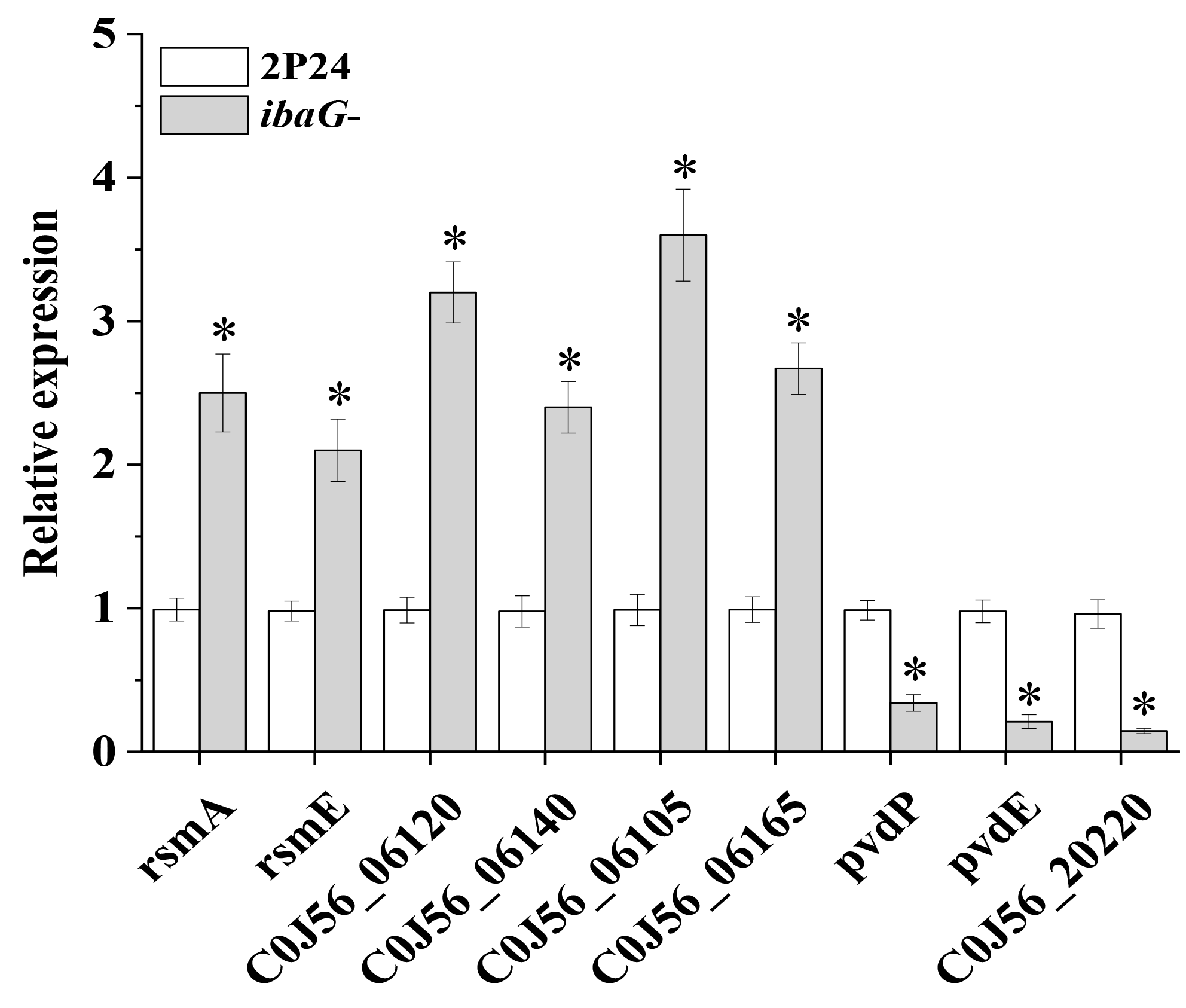


Figure S4. The expression of *rsmA*, *rsmE*, C0J56_06120, C0J56_06140, C0J56_06105, C0J56_06165, *pvdP*, *pvdE*, and C0J56_20220 were regulated by IbaG. The mRNA levels of these genes were measured by RT-qPCR. The experiments were carried out in triplicate with three independent RNA samples and the mean values ± standard deviations are indicated. * indicates *P* < 0.05.

**
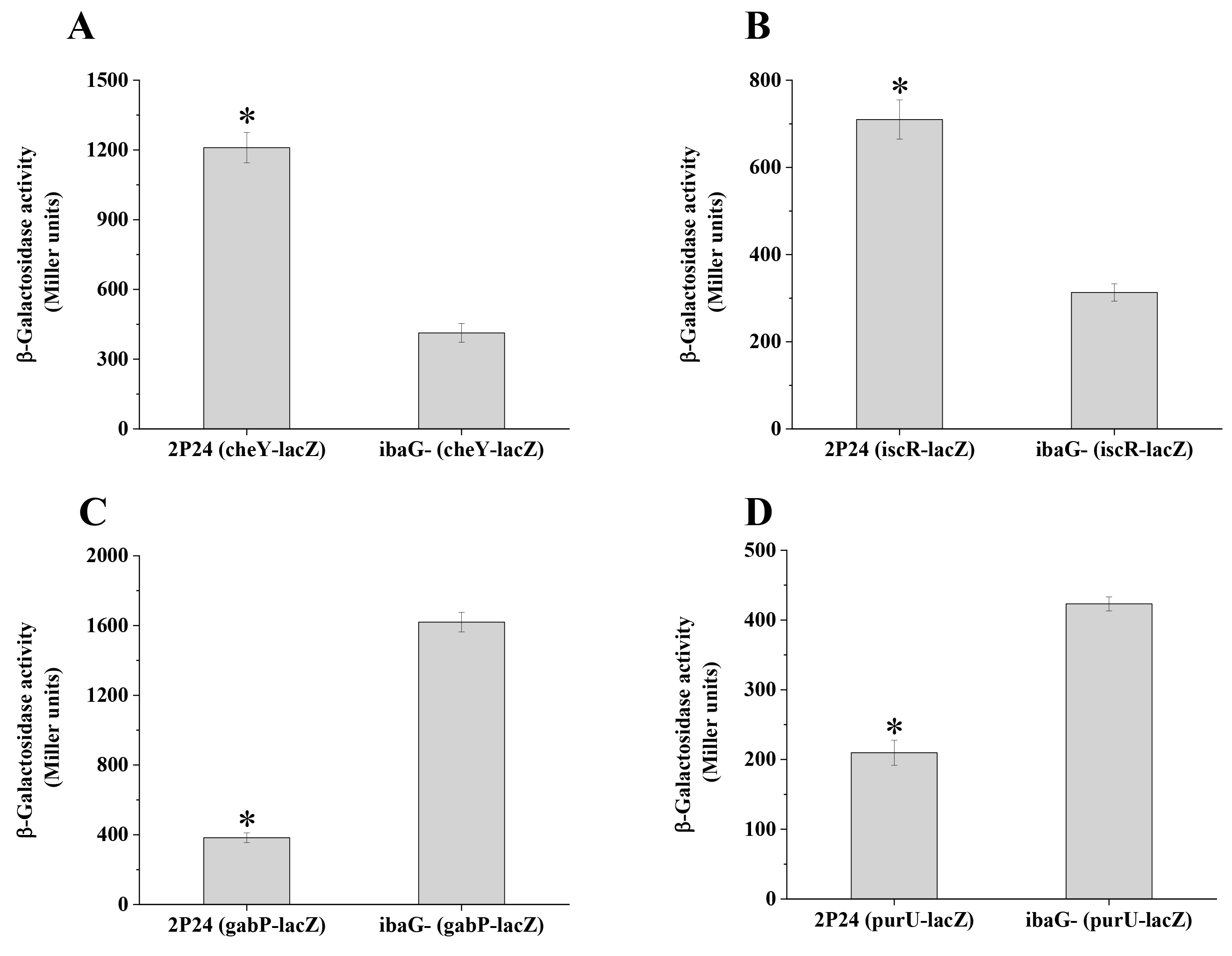
**

Figure S5. The expression of *cheY*, *iscR*, *gabP*, and *purU* is regulated by IbaG in *P. fluorescens* 2P24. β-Galactosidase activities of the transcriptional fusions *cheY*-*lacZ* (A), *iscR*-*lacZ* (B), *gabP*-*lacZ* (C), and *purU*-*lacZ* (D) were determined in the wild type strain 2P24 and its *ibaG* mutant. All experiments were performed in triplicate, and the mean values ± standard deviations are indicated. * indicates *P* < 0.05.


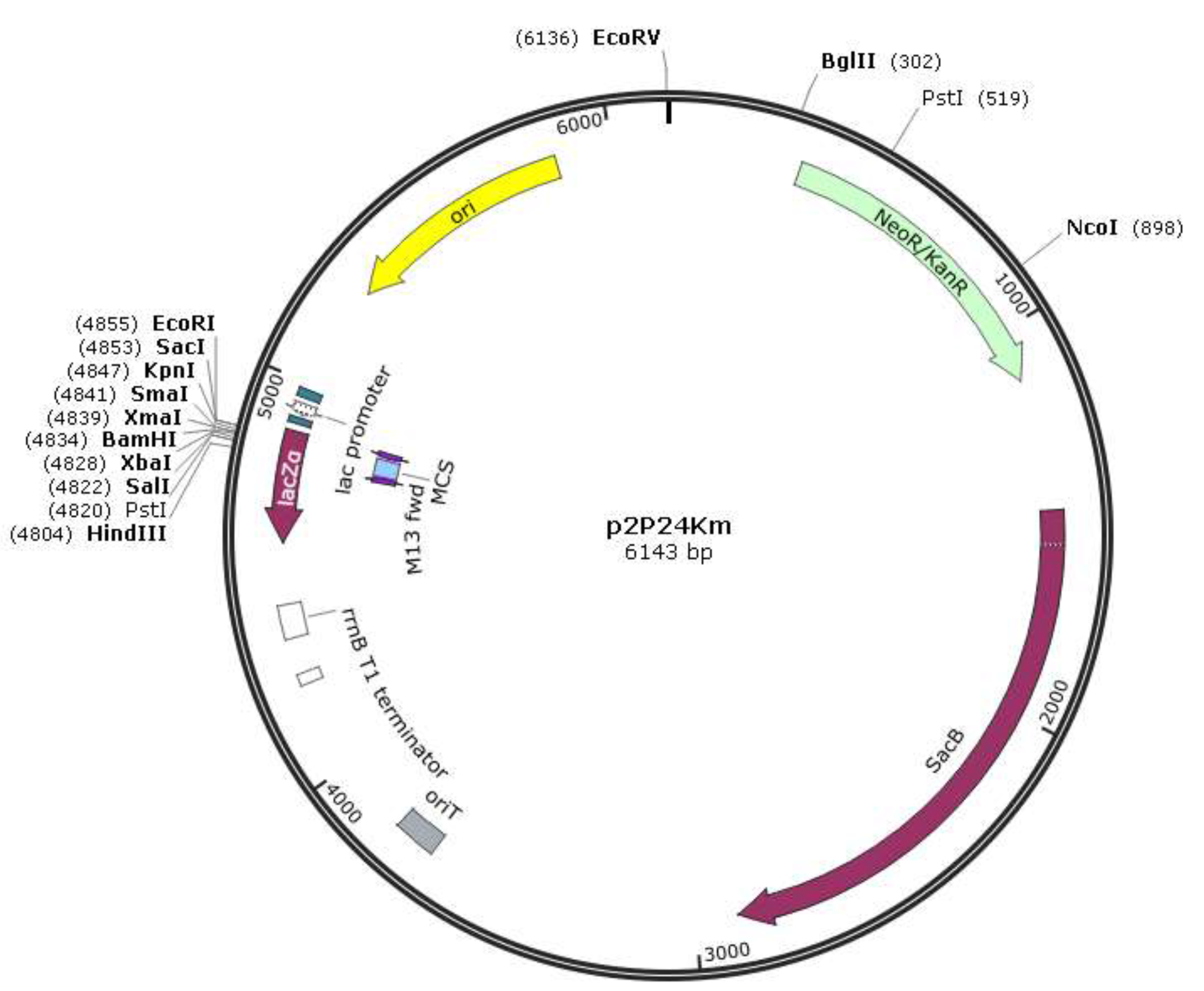


Figure S6. The map of plasmid p2P24Km. The 6143 bp plasmid contains a Kanamycin resistance gene (neo^R^/Kam^R^) and a *sacB* gene which facilitate the screening of the deletion mutants with the presence of the kanamycin antibiotic or the sucrose substrate. The two DNA fragments that flank the upstream and downstream regions of the target gene, such as *ibaG* or *bolA*, were cloned into the restriction enzyme sites as described in the Methods.


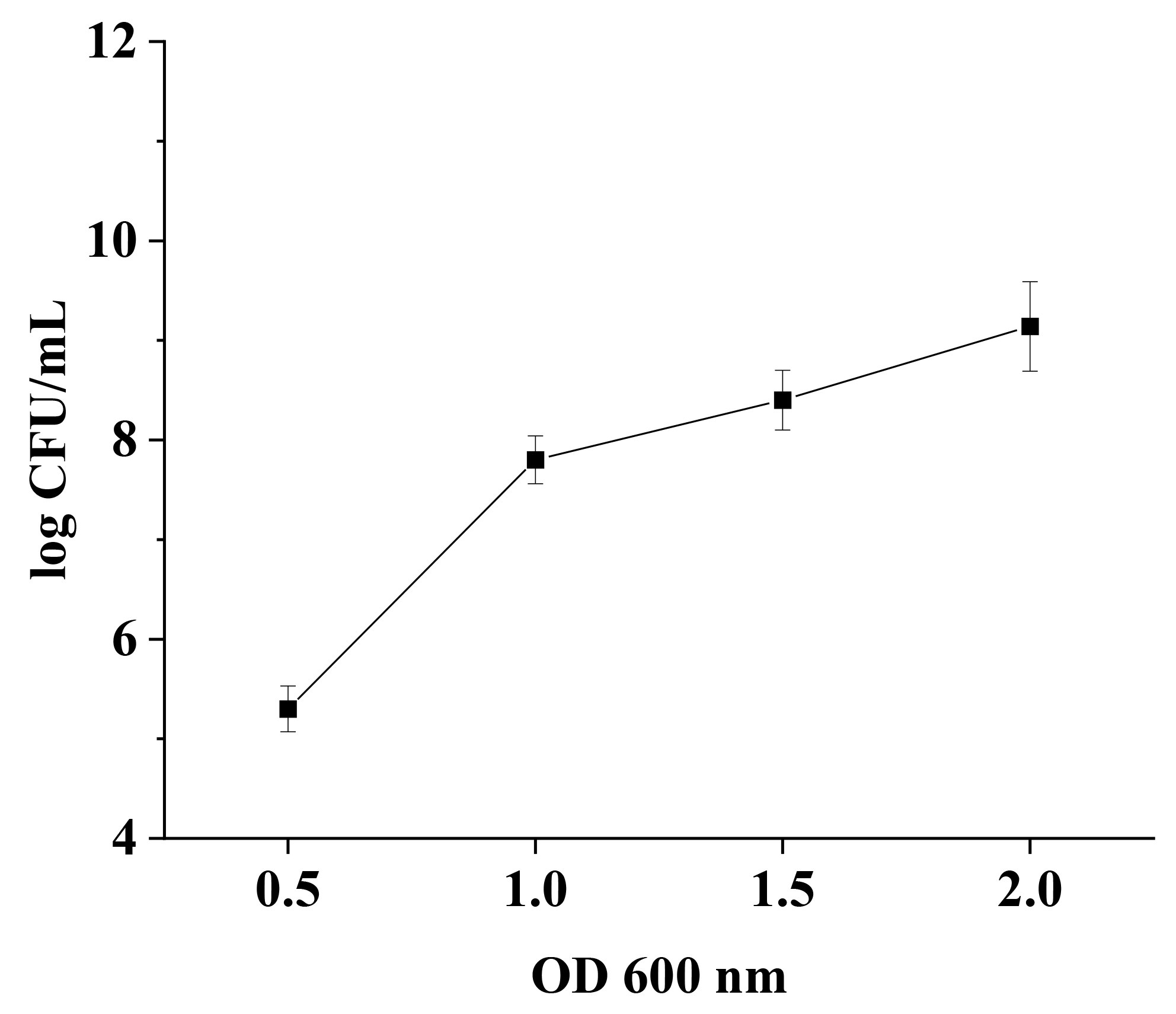


Figure S7. The correlation of the optical density (OD) at 600 nm and the colony-forming units (CFU) of strain 2P24. Strain 2P24 was cultured in LB medium and the CFU was determined at different OD 600 nm. The experiments were performed in triplicate and the mean values ± standard deviations are indicated.
